# Multi-omic analysis reveals that *Bacillus licheniformis* enhances pekin ducks growth performance via lipid metabolism regulation

**DOI:** 10.3389/fphar.2024.1412231

**Published:** 2024-06-11

**Authors:** Lei Li, Liangyu Yang, Limei Zhang, Fengping He, Zhaofei Xia, Bin Xiang

**Affiliations:** ^1^ College of Animal Veterinary Medicine, Yunnan Agricultural University, Kunming, China; ^2^ College of Animal Veterinary Medicine, China Agricultural University, Beijing, China

**Keywords:** *Bacillus* licheniformis, pekin ducks, lipid metabolism, transcriptomics, multi-omics

## Abstract

**Introduction:** *Bacillus licheniformis* (*B.licheniformis*) was widely used in poultry feeds. However, it is still unclear about how B.licheniformis regulates the growth and development of Pekin ducks.

**Methods:** The experiment was designed to clarify the effect and molecular mechanism of *B. licheniformis* on the lipid metabolism and developmental growth of Pekin ducks through multiomics analysis, including transcriptomic and metabolomic analyses.

**Results:** The results showed that compared with the control group, the addition of 400 mg/kg *B. licheniformis* could significantly increase the body weight of Pekin ducks and the content of triglyceride (p < 0.05), at the same time, the addition of *B. licheniformis* could affect the lipid metabolism of liver in Pekin ducks, and the addition of 400 mg/kg *B. licheniformis* could significantly increase the content of lipoprotein lipase in liver of Pekin ducks. Transcriptomic analysis revealed that the addition of *B. licheniformis* primarily impacted fatty acid and glutathione, amino acid metabolism, fatty acid degradation, as well as biosynthesis and elongation of unsaturated fatty acids. Metabolomic analysis indicated that *B. licheniformis* primarily affected the regulation of glycerol phospholipids, fatty acids, and glycerol metabolites. Multiomics analysis demonstrated that the addition of B. licheniformis to the diet of Pekin ducks enhanced the regulation of enzymes involved in fat synthesis via the PPAR signaling pathway, actively participating in fat synthesis and fatty acid transport.

**Discussion:** We found that *B. licheniformis* effectively influences fat content and lipid metabolism by modulating lipid metabolism-associated enzymes in the liver. Ultimately, this study contributes to our understanding of how *B. licheniformis* can improve the growth performance of Pekin ducks, particularly in terms of fat deposition, thereby providing a theoretical foundation for its practical application.

**Conclusion:** *B. licheniformis* can increase the regulation of enzymes related to fat synthesis through PPAR signal pathway, and actively participate in liver fat synthesis and fatty acid transport, thus changing the lipid metabolism of Pekin ducks, mainly in the regulation of glycerol phospholipids, fatty acids and glycerol lipid metabolites.

## Background

In addition to plant extracts, organic acids, polysaccharides and vaccines, probiotics promote animal growth (Abudabos et al., 2016; Mingmongkolchai and Panbangred, 2018). *Bacillus licheniformis* (*B.licheniformis*) is a Gram-positive bacterium that exhibits resistance to high-temperature, acid and alkali, and high-stress conditions (Elisashvili et al., 2019). *B. licheniformis* produces digestive enzymes, lysozymes, bacteriocins, antimicrobial peptides, and other bioactive substances that can improve animal production performance by improving feed digestibility, stimulate immune system development, and enhance intestinal mucosal barrier function (Gopi et al., 2022; Li et al., 2022; Ramirez-Olea et al., 2022; Todorov et al., 2022). *B. licheniformis* protects from potentially harmful intestinal microorganisms and inhibits pathogenic bacteria colonization, promotes the proliferation of potentially beneficial microorganisms, and maintains intestinal microbiota homeostasis (Zhou et al., 2016). In addition, *Bacillus*-based probiotics enhance broiler intestinal mucosal immune function by increasing the expression of TLRs, related downstream junction proteins, and NF-κB mRNA (Rajput et al., 2017). Dietary administration of *B. licheniformis* regulates the composition and structure of the intestinal microbiota in broilers challenged with Necrotic enteritis (Xu et al., 2018).

Transcriptomic sequencing is a high-throughput technique used to sequence RNA and create expression maps (Sklavenitis-Pistofidis et al., 2021). This approach allows for sample RNA identification and annotation, as well as functional enrichment, cluster analysis, and information mining, using databases such as Gene Ontology (GO), Clusters of Orthologous Groups (COG), and Kyoto Encyclopedia of Genes and Genomes (KEGG). Transcriptomic sequencing is highly sensitive, specific, and systematic, making it a valuable tool in biomedical research for the identification of molecular mechanisms underlying disease initiation and development (Kuppe et al., 2021; Sklavenitis-Pistofidis et al., 2021; Su et al., 2022). RNA sequencing is a high-throughput quantitative technique used to study gene expression and is important for the advancement of livestock genomics. In recent years, the cost of high-throughput sequencing technologies has decreased significantly, increasing the number of RNA-Seq studies in the livestock sector. However, compared with other fields, RNA-Seq studies in livestock, particularly poultry, are relatively limited, and this requires further investigation (Keel and Lindholm-Perry, 2022).

Metabolites are end-products or intermediates of the metabolic processes and are closely related to an organism’s phenotype, offering an innovative approach to directly and effectively monitor biological processes and mechanisms of metabolism (Chung et al., 2018; Sinem et al., 2019). Metabolomics enables qualitative and quantitative analysis of metabolites, making it a valuable tool for studying the metabolomic phenotypes of different individuals (Aszyk et al., 2018). It can be used to examine metabolic pathways and corresponding metabolites influenced by factors, such as diseases or drugs, as well as to assess the safety of food and drugs (Klassen et al., 2017). Metabolomics allows for the identification and quantification of characteristic metabolites of biological significance, thereby elucidating biological processes and metabolic mechanisms (Wang et al., 2010; Zhang et al., 2016).

Genomics, transcriptomics, and metabolomics are valuable tools for studying animal growth processes at different levels (Klassen et al., 2017; Sinem et al., 2019; Keel and Lindholm-Perry, 2022; Sah et al., 2022). However, single-omics studies only provide information on changes at a single level and fail to capture the interconnections between complex biological processes (Wu et al., 2021). In this study, using Illumina-based RNA-seq technology, we aimed to determine the impact of *B. licheniformis* on gene regulation in Pekin ducks. Through bioinformatics analysis, we aimed to identify differentially expressed genes and related signaling pathways and sought to elucidate the underlying molecular regulatory mechanisms. Additionally, by conducting non-targeted metabolomics, we aimed to evaluate the metabolic processes of Pekin ducks fed a *B. licheniformis*-containing diet. By combining KEGG functional annotation and enrichment analysis, we aimed to determine the relationship between the transcriptomic and metabolomic and to systematically examine the fatty regulatory mechanisms.

## Materials and methods

### Animal ethics

All animal procedures used in this study were conducted in accordance with the Guidelines for the Care and Use of Laboratory Animals of China Agricultural University, and the experimental design was approved by the Animal Care and Use Committee of China Agricultural University (Beijing, China).

### Prepare of *Bacillus licheniformis*

The *B. licheniformis* (*B*.*licheniformis*) was provided by Guangzhou Weiyuan Biotechnology Co., Ltd., and the number of viable bacteria was 1.0 × 10^10^ CFU/g. The production lot number is WYYLRSJ100. The experimental animal Pekin ducks were provided by Tianjin Poultry Breeding Professional Cooperative.

### Experimental design and sample collection

One-day-old healthy Pekin ducks (*n* = 360) with similar initial body weight (60 ± 0.5 g) were included in the study. The ducks were randomly divided into four groups with six replicates per group and 15 ducks per replicate. The control group (CON) was fed a corn-soybean meal basal diet. The experimental groups were fed the basal diet supplemented with different concentrations of *B. licheniformis*: 200 mg/kg (LLB), 400 mg/kg (MLB), and 800 mg/kg (HLB). The probiotic strain, *B. licheniformis* used in this study was obtained from Guangzhou Weiyuan Biotechnology Co., Ltd. Before experiment initiation, the duck houses were thoroughly cleaned and disinfected. Pekin ducks were raised with unrestricted access to drinking water and food and were exposed to 23 h of light. For the first week, the duck house was maintained at 35°C and gradually reduced to 25°C (maintained with automated equipment). The ducks were fed twice daily at 8:00 and 16:00. Routine immunization procedures were followed, feces was removed daily at 7:00, and the sink and trough were cleaned weekly. On day 42, jejunum and cecal tissue, blood, and liver tissue samples were collected from each group and stored at −80°C. In this study, Pekin ducks were euthanized using CO_2_ asphyxiation. Before the ducks were put in, 40% concentration CO_2_ was injected into the euthanasia box for 30–40 s, then CO_2_ was turned off, and the Pekin ducks were put in. Reperfusion of 80% concentration of carbon dioxide in the box for 5 min, after confirming that the Pekin ducks did not move, did not breathe, and the pupil was dilated, the carbon dioxide was turned off, and the animal was observed for another 3 min to confirm the death of Pekin ducks.

### Growth performance and serum biochemistry analysis

The body weight and food intake of the Pekin ducks were recorded daily. Collected blood samples were centrifuged at 3,000 *g* and 4°C for 20 min and the obtained serum was stored at −20°C until further analysis. Serum total cholesterol (TC), triglyceride (TG), high-density lipoprotein cholesterol (HDL-C), low-density lipoprotein cholesterol (LDL-C), and free fatty acid (FFA) levels were detected directly using biochemical instruments. Very low-density lipoprotein (VLDL) levels were measured using ELISA Kit (Enzyme-linked Biotechnology Co., Ltd, Shanghai, China). Levels of fatty acid synthetase (FAS), acetyl-CoA carboxylase (ACC), malate enzyme (ME) and lipoprotein lipase (LPL) were determined in liver tissues using the Duck ELISA Kit (Enzyme-linked Biotechnology Co., Ltd, Shanghai, China), following the manufacturer’s instructions.

### Mutiomics analysis

In a previous study, we found that the addition of 800 mg/kg *B licheniformis* to the diet of Pekin ducks improved growth performance; therefore, we performed multiomics analysis to determine the mechanisms underlying the role of *B. licheniformis* in improving growth performance.

### Untargeted metabolomics assays

Untargeted metabolomics analysis was performed on blood samples of Pekin ducks. High-resolution mass spectrometry was performed using the Waters Acquity I-Class PLUS and Xevo G2-XS QT spectrometers with an Acquity UPLC HSS T3 column and the positive and negative ion modes with mobile phases. Principal component analysis (PCA) provides a preliminary understanding of the overall metabolic differences between samples and the degree of variability between samples within a group; therefore, we performed PCA analysis in both ion modes, with the principal component score plots in positive and negative ion modes. Positive ion mode: mobile phase A, 0.1% formic acid aqueous solution; mobile phase B, 0.1% formic acid acetonitrile; negative ion mode: mobile phase A, 0.1% formic acid aqueous solution; mobile phase B, 0.1% formic acid acetonitrile. For metabolite detection, samples (1 µL) were directly injected into the spectrometer and the liquid chromatography gradient parameters are listed in Supplementary Table S1, additional Supplementary File S1.

Mass spectrometry conditions: Primary and secondary mass spectrometry data was acquired using a Waters Xevo G2-XS QTof high-resolution mass spectrometer with MSe mode using acquisition software (MassLynx V4.2, Waters). Both low and high collision energies were acquired simultaneously using the dual-channel data acquisition mode. The low collision energy was 2 V, high collision energy interval 10–40 V, and scanning frequency 0.2 s for one mass spectrogram. MassLynx V4.2 was used to collect the raw data. The acquired data was processed using Progenesis QI software where peak extraction and peak alignment were performed based on the Progenesis QI software online METLIN database and Bemac’s self-constructed library. Theoretical fragmentation identification was performed, simultaneously, to ensure that the quality deviation was controlled at less than 100 ppm.

### Transcriptomic analysis

Total RNA was extracted from liver tissue using Trizol, according to the manufacturer’s instructions. The extracted RNA was quantified and the purity was detected using a Nanodrop (OD260/OD280 between 1.7 and 2.5 was considered acceptable). Nanodrop, Qubit 2.0, Agilent 2,100 and electrophoresis (on a 1.2% agarose gel) were used to detect the purity, concentration, and integrity of RNA samples, respectively. Next, three samples were selected from each treatment group for library construction and the effective concentration of the library was accurately quantified by qPCR. Subsequently, high-throughput sequencing was performed using NovaSeq 6,000 with the PE150 mode. The filtered high-quality reads were randomly selected and the reference genomes of mallards and crested ducks were compared using the BLAST software. Fragments Per Kilobase of exon model per million mapped fragments (FPKM) was used to measure the level and distribution of gene expression. The Spearman’s rank coefficient was used to evaluate biological replicate correlation. Differential expression analysis between the five treatment groups was performed using edgeR software. Differentially expressed genes (DEGs) were identified by |Fold Change|≥1.5 and *p* < 0.05. An MA plot was used to visually represent the overall distribution of gene expression among the groups. Functional annotation of DEGs was performed by Gene Ontology (GO), Cluster of Orthologous Groups of proteins (COG), and Kyoto Encyclopedia of Genes and Genomes (KEGG) analysis.

### Integrated analysis of metabolomics and transcriptomics analysis

Differential metabolites were analyzed at the phylum and genus levels of the microorganisms. Pearson’s correlation analysis was used to determine the relationship between differential metabolites and differential gut microbiota (|CC| > 0.06 and *p* < 0.05). The DEGs were then functionally annotated using the GO and KEGG enrichment analysis. To better define the transcriptional regulation mechanisms of metabolic pathways, DEGs and differentially expressed metabolites (DEMs) were extracted from each group, and correlations between all genes and metabolites were determined using Pearson’s correlation (|CC| > 0.80 and CCP <0.05). Simultaneously, differential genes and metabolites of the same group were mapped to the KEGG pathway to identify their relationships with pathways, genes, and metabolites.

### Verification of differentially expressed genes by qRT-PCR

The identified DEGs were verified by fluorescent quantitative PCR, and β-actin RNA was used as an internal reference. Total uterine RNA was extracted using the RNA Easy Fast tiss/Cell kit DP451 (Tiangen Biochemical Technology Co., Ltd., Beijing, China), and the quality of RNA was determined using the OD260/280 and OD260/230 ratios. The PCR reaction was detailed in Supplementary Table S2 and the primer sequences were shown in Supplementary Table S3, additional file1. Amplification was performed as follows: pre-denaturation at 95°C for 30 s and 35 cycles of denaturation at 95°C for 10 s, annealing at 60°C for 30 s, and extension at 95°C for 10 s.

### Statistical analysis

Excel 2010 was used to process test data. Next, one-way ANOVA (LSD) was performed using IBM SPSS 26.0 analysis software, and Duncan’s test was used for multiple comparison tests. Data are represented as mean ± standard error. Statistical significance was set at *p* < 0.05.

## Results

### Growth performance and serum biochemistry analysis

**FIGURE 1:**
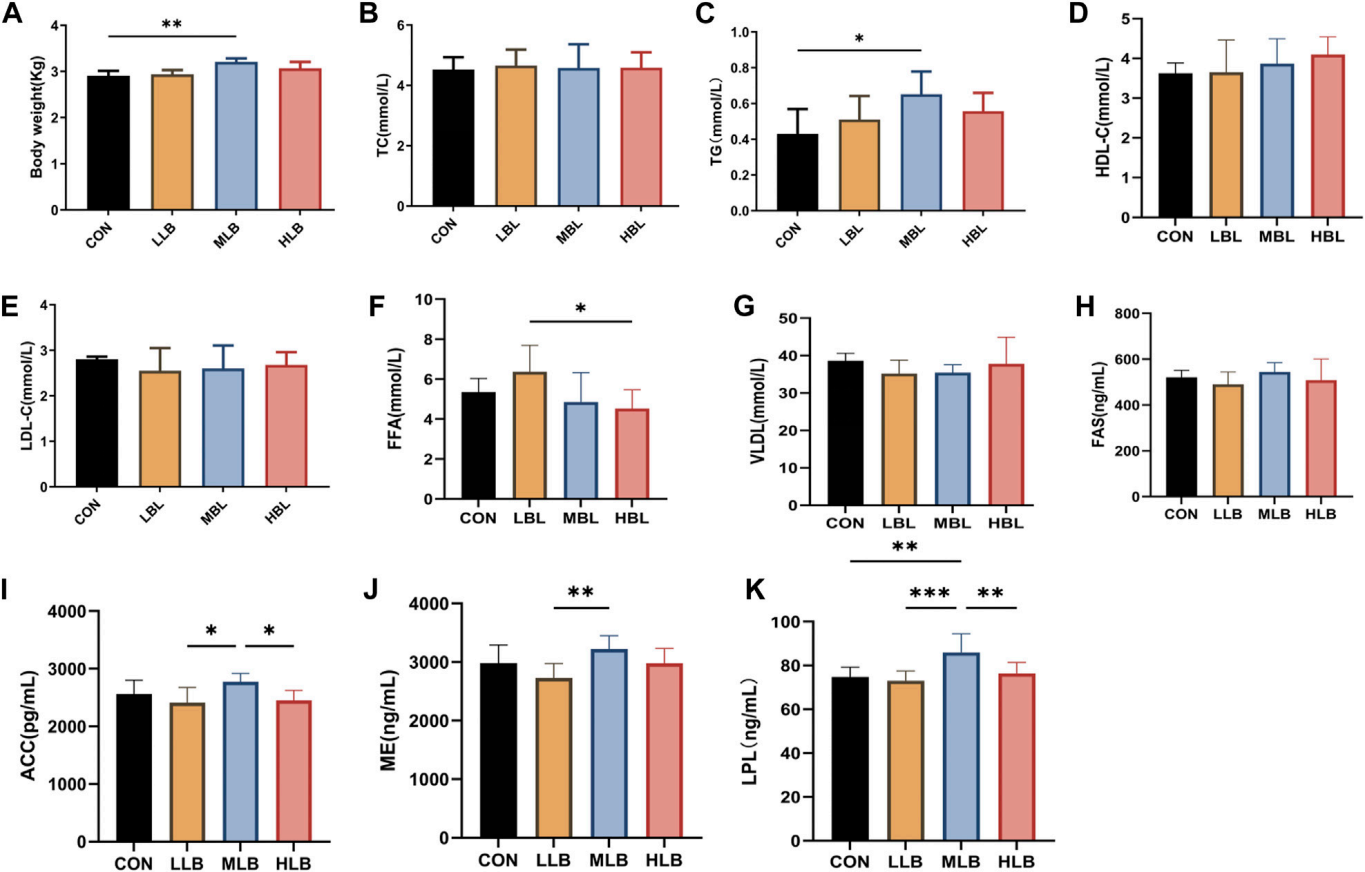
Efects of *Bacillus licheniformis* on growth performance, blood lipid levels and liver lipid metabolism of Pekin ducks. **(A)** Growth performance of Pekin ducks. **(B–F)** Blood lipid levels. **(G–K)** Liver lipid metabolism. Values were means and standard errors, * indicate signifcant diferences among diferent groups (*p < 0.05*), **indicate signifcant diferences among diferent groups (*p < 0.01*), *** indicate signifcant diferences among diferent groups (*p < 0.001*). CON, Basal diet; LLB, Basal diet+200 mg/kg *Bacillus licheniformis*; MLB, Basal diet+400 mg/kg *Bacillus licheniformis*; HLB, Basal diet+800 mg/kg *Bacillus licheniformis*.

The effects of *B. licheniformis* supplementation on the growth performance of Pekin ducks are shown in Figure 1A. On Day 42, the body weight of Pekin ducks in the MLB group was significantly higher than that of the CON group (*p* < 0.05). In addition, *B. licheniformis* affected serum TG, TC, LDL-C, HDL-C, and VLDL; TG in the MLB group was significantly higher than that of the CON group (*p* < 0.05; Figures 1B–F).

### Lipid metabolism in liver

The liver lipid metabolism of Pekin ducks was analyzed (Figures 1G–K). Compared with the HLB group, the FFA content was significantly higher in the LLB group (*p* < 0.05). Compared with the CON group, the dietary addition of *B. licheniformis* caused an increase in liver fatty acid synthase (FAS); however, the difference was not significant. Compared with LLB group, ACC, ME and LPL in the liver of MLB group was significantly higher (*p* < 0.05). Compared with the CON group, LPL was significantly higher in the MLB group (*p* < 0.01).

### Serum untarget metabolites analysis

**FIGURE 2:**
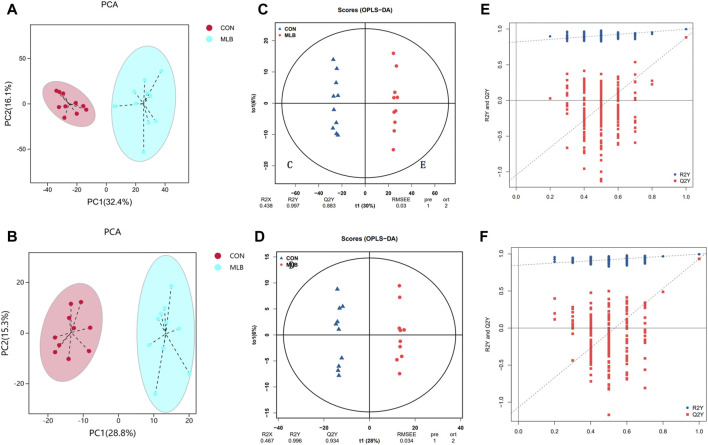
PCA, OPLS-DA scores and Permutation test of serum metabolites in positive and negative ion mode between with CON and MLB. **(A,C,E)** PCA, OPLS-DA scores and Permutation test in positive ion mode. **(B,D,F)** PCA, OPLS-DA scores and Permutation test in negative ion mode. CON, Basal diet; MLB, Basal diet + 400 mg/kg *Bacillus licheniformis*.

Qualitative and quantitative metabolomic analyses was performed on 20 samples. A total of 2,999 metabolites were detected, 2,300 and 699 in the positive and negative ion modes, respectively (Figures 2A,B). OPLS-DA was used to analyze the degree of correlation between the CON and MLB groups. The OPLS-DA score plots of the serum samples in the positive ion mode are shown in Figure 2C. R2X, R2Y, and Q2Y were 0.438, 0.997, and 0.883, respectively. The OPLS-DA score plots of the serum samples in the negative ion mode are shown in Figure 2D. R2X, R2Y, and Q2Y were 0.467, 0.996, and 0.934, respectively. These values indicate that the analytical model had good prediction abilities.Figures 2E,F shows the displacement test plots in the positive and negative ion modes, respectively. Both R2Y and Q2Y were smaller than the R2Y and Q2Y values of the original model, which indicated that the model was meaningful and could be used for differential metabolite screening.

**FIGURE 3:**
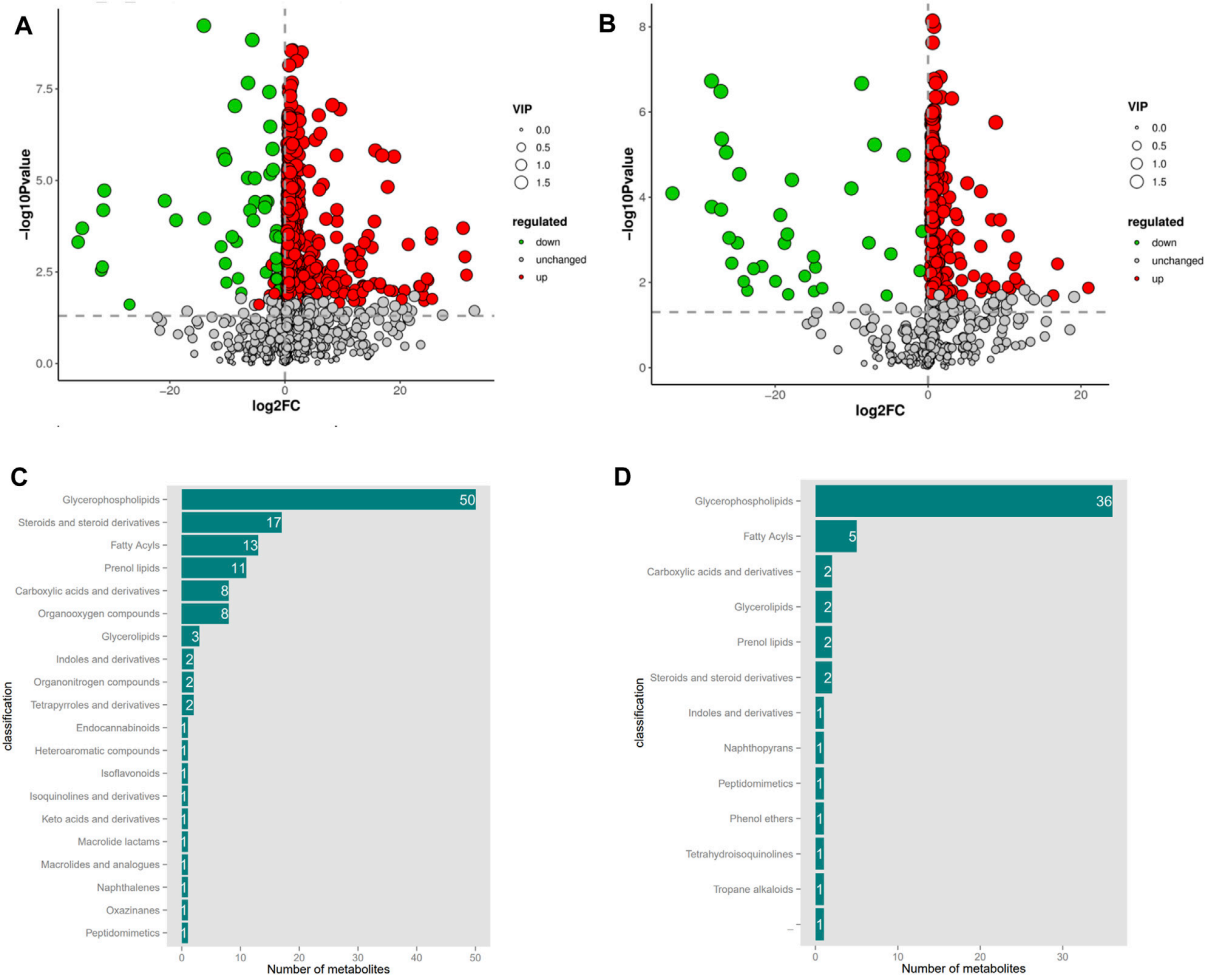
Volcanic map and Classification of serum differential metabolites in positive and negative ion mode between with CON and MLB. **(A,B)** Volcanic map in positive and negative ion mode; **(C,D)** Classification of serum differential metabolites in positive and negative ion mode. CON, Basal diet; MLB, Basal diet+400 mg/kg *Bacillus licheniformis*.

Based on the OPLS-DA model, differential metabolite screening was performed by combining the multiplicity of differences, *t*-test, and VIP value. The screening criteria was as follows: FC > 1, *p* < 0.05, and VIP>1. Significant DEMs were identified; 1,033 in the positive ion mode (956 upregulated and 77 downregulated) and 279 in the negative ion mode (243 upregulated and 36 downregulated). DEM enrichment analysis was performed using the HMDB database (Figures 3A,B). Metabolite classification maps for the positive and negative ion modes are shown in Figures 3C,D, respectively.

In the positive ion mode, the vast majority of DEMs were annotated to lipid metabolism pathways, among which 50 were annotated to glycerophospholipids, 17 to steroids and steroid derivatives, 13 to fatty acids, 11 to pregnenolone lipids, three to glycerolipids, and eight to carboxylic acids and derivatives. In the negative ion mode, the majority of DEMs were annotated in the lipid metabolic pathway, of which 36 were annotated in glycerophospholipids, five in fatty acids, and two in glycerolipids. The trends in metabolite changes were comparable in both positive and negative ion modes; the total categories were glycerophospholipids, fatty acids, and glycerolipids. This indicated that the addition of *B. licheniformis* to Pekin duck feed significantly altered the glycerophospholipid, fatty acid, and glycerolipid metabolites in Pekin ducks, thereby affecting lipid metabolism.

### Liver transcriptome analysis

**FIGURE 4:**
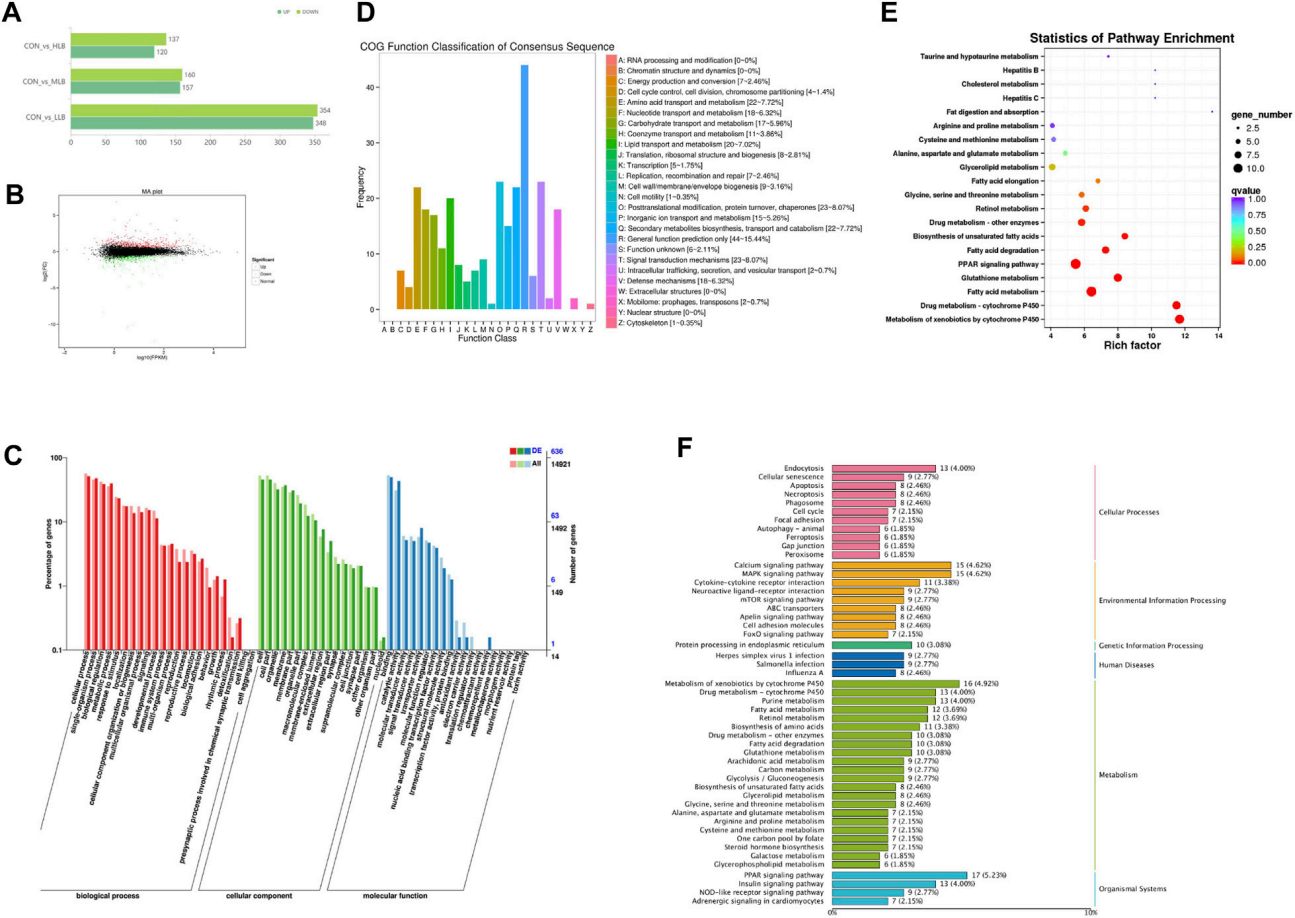
Efects of *Bacillus licheniformis* on the serum metabolites of Pekin ducks. **(A)** Numbers of differently expressed genes (DEGs). **(B)** MA map of CON and MLB differently expressed genes. **(C)** Gene ontology (GO) enrichment analysis of DEGs. **(D)** Cluster of Orthologous Groups (COG) analysis of DEGs. **(E)** Statistics of KEGG pathway enrichment by diferent metabolites [log2(FC) ≥1.5 and *p* < 0.01]. The size of the dots indicates the number of diferentially abundant metabolites and the color of the dots represents the *p*-value. **(F)** KEGG signaling pathways enriched by diferent metabolites. CON, Basal diet; MLB, Basal diet+400 mg/kg *Bacillus licheniformis*.

On Day 42, 116.48 Gb of clean sequences were obtained from 15 samples, and each sample scored an average sequence of 6.23 Gb. Compared with the CON group, 472 (235 upregulated and 237 downregulated), 357 (217 upregulated and 140 downregulated), and 67 (37 upregulated and 30 downregulated) DEGs were identified in the LLB, MLB, and HLB groups, respectively (Figure 4A). Based on the data obtained by RNA-seq sequencing, the expression of differential genes between the samples of the CON group and the MCB group was analyzed in detail, log2(FC) ≥1.5 and *p*-value <0.01 were used as the screening criteria, and the genes with gene expression levels greater than the expression threshold of one in the same group were statistically analyzed and MA plots were drawn, and the results showed that a total of 848 differentially expressed genes were screened between the two groups, of which 479 genes were upregulated and 369 genes were downregulated (Figure 4B).

GO analysis assigned DEGs into three categories: biological processes, cellular components, and molecular functions (Figure 4C). In biological processes, DEGs were primarily related to cell metabolism, bioregulation, and metabolic processes. In cellular components, DEGs were associated with organelles, cell membranes, and macromolecular complexes. In molecular functions, DEGs were mainly concentrated in catalysis, transport, and antioxidant activity. COG analysis revealed that the DEGs were primarily distributed in general function prediction and transport and metabolism of amino acids, lipids, nucleotides, and carbohydrates (Figure 4D). KEGG enrichment analysis showed that the metabolic pathways were mainly enriched in nutrient metabolism, such as fatty acid and glutathione metabolism, fatty acid degradation, biosynthesis of unsaturated fatty acids, fatty acid elongation, as well as glycine, serine, and threonine metabolism (Figure 4E; Figure 3F).

## Combined metabolome and transcriptome analysis

### Multivariate analysis

**FIGURE 5:**
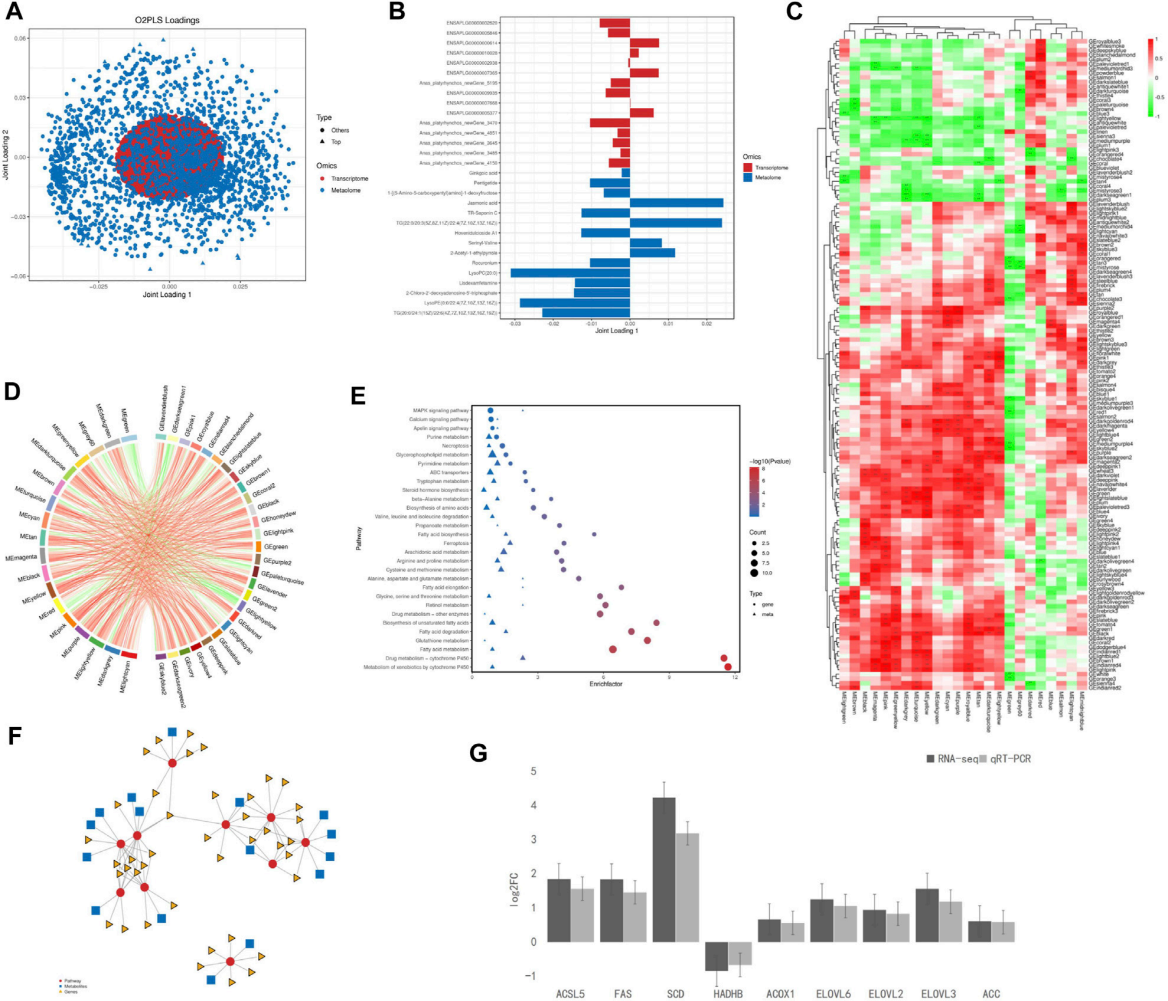
Combined multi-omics analysis. **(A)** O2PLS loading label analysis between significantly differential genes in the liver and differential metabolites in the serum; **(B)** Histogram of significantly differential genes in the liver and differential metabolites in the serum; **(C)** WGCNA Heat maps of significantly differential genes in the liver and differential metabolites in the serum; Red indicates a positive correlation; the green indicates a negative correlation. ***, **, and * represent levels of significance (*p* < 0.001, *p* < 0.01, and *p* < 0.05, respectively); **(D)** Chord label of significantly differential genes in the liver and diferential metabolites in the serum; **(E)** Bubble map of significantly differential genes in the liver and diferential metabolites in the serum; **(F)** Network of significantly differential genes in the liver and diferential metabolites in the serum; **(G)** Verification of significant differently expressed genes in liver by RT-PCR.

The O2PLS loading no-label revealed a correlation between the data sets, metabolomics was assigned more weight than transcriptomics (Figure 5A). Based on the relationship between the two data matrices, we selected the genes and metabolites with the top 15 length load values in the first two dimensions (with the highest degree of association) and generated bar charts (Figure 5B). The located metabolites were Ginkgoic acid, Pentigetide, 1-[(5-Amino-5-carboxypentyl)amino]-1-deoxyfructose, Jasmonic acid, TR-Saponin C, TG [22:0/20:3 (5Z, 8Z, 11Z)/22:4 (7Z,10Z,13Z, 16Z)], Hovenidulcioside A1, Serinyl-Valine2-Acetyl-1-ethylpyrrole, Rocuronium, LysoPC (20:0), Lisdexamfetamine, 2- Chloro-2′-deoxyadenosine- 5′-triphosphate, LysoPE [0:0/22:4 (7Z,10Z,13Z, 16Z)] and TG [20:0/24:1 (15Z)/22:6 (4Z,7Z,10Z,13Z,16Z, 19Z)].

### Weighted gene co-expression network analysis (WGCNA)

WGCNA dimensionality reduction analysis was performed on the transcriptome and metabolome datasets, and cluster analysis was performed using a color gradient (Figure 5C, green indicates a negative correlation and red indicates a positive correlation). WGNCA of transcriptomic and metabolic data resulted in 144 and 24 modules, respectively. Most of the data modules within the two groups correlated positively; however, the MEgreen module in the metabolite group negatively correlated with most of the modules in the transcriptome group.

The correlation chord plots showed that 18 metabolite modules were selected among the top 30 absolute values of the correlation coefficients, and each metabolite module strongly correlated with the 27 labeled gene modules: MEgreen, MEgrey60, MElightcyan, MEred, MEdarkgreen, MEcyan, MEpurple, MEblack, MEmagenta, MEdarkturquoise, MElightyellow, MEpink, MEdarkgrey, MEtan, MEturquoise, MEyellow, MEbrown, and MEgreenyellow (Figure 5D).

### Comparative analysis of KEGG pathways

The enriched pathways were analyzed for the DEGs identified by transcriptomics and metabolomics. 66 significantly enriched pathways were identified and the top 30 pathways with the largest number of metabolites were mapped (Figure 5E). The DEGs were mainly enriched in the metabolic effects of cytochrome P450 on xenobiotics; drug metabolism-cytochrome P450; fat digestion and absorption; cholesterol metabolism; unsaturated fatty acid biosynthesis; glutathione metabolism; taurine and hypotaurine metabolism; fatty acid degradation; fatty acid elongation; glycine, serine, and threonine metabolism; and fatty acid metabolism. Differential metabolites were mainly enriched in drug metabolism-cytochrome P450; retinol metabolism; fatty acid elongation; alanine, glycine, serine, and threonine metabolism; fat digestion and absorption; ketone body synthesis and degradation; and MAPK signaling pathways. Significantly enriched pathways shared by both datasets were drug metabolism-cytochrome P450; fatty acid elongation; fat digestion and uptake; and glycine, serine, and threonine metabolism.

### KEGG pathway xml analysis

KGML (KEGG Markup Language) revealed that (S)-3-Hydroxytetradecanoyl-CoA was involved in fatty acid degradation, metabolism, and elongation pathways; L-palmitoyl carnitine in fatty acid degradation and metabolism pathways; ENSAPLG00000002680 in drug metabolism-cytochrome P450, fatty acid degradation, glutathione and retinol metabolism, metabolic effects of cytochrome P450 on xenobiotics, and drug metabolism; ENSAPLG00000007893 in fatty acid degradation and metabolism; ENSAPLG00000016081 in fatty acid degradation and metabolism and unsaturated fatty acid biosynthesis; and ENSAPLG00000012370, ENSAPLG00000002641, and ENSAPLG 00000011982 in fatty acid metabolism and elongation and unsaturated fatty acid biosynthesis (Figure 5F).

### Liver transcriptome fluorescence PCR validation

Six pathways were significantly enriched in lipid metabolism: fatty acid degradation, biosynthesis of unsaturated fatty acids, fatty acid elongation, glycerolipid metabolism, fatty acid biosynthesis, and arachidonic acid metabolism. Nine DEGs, significantly related to lipid metabolism (ACSL5, FAS, SCD, LPL, HADHB, ACOX1, ELOVL2, ELOVL3, and ELOVL6) were identified in the liver. The expression of these genes was further verified by real-time RT-qPCR using the primer sequences listed in Schedule A-5 (Figure 5G). Among the identified DEGs, ACSL5, FAS, SCD, ACOX1, ELOVL2, ELOVL3, ELOVL6, and ACC were upregulated and HADHB was downregulated.

## Discussion

*Bacillus* is one of the most widely used probiotic species in feed and farming, and *B. licheniformis* improves the growth performance of chickens (Abudabos et al., 2016; Bernardeau et al., 2017; Bai et al., 2018; Dong et al., 2020; Ramlucken et al., 2020). However, the mechanism underlying the role of *B. licheniformis* in enhancing growth performance remains unclear. In this study, we aimed to investigate the effect of *B. licheniformis* on the growth performance of Pekin ducks and, through the use of multi-omics technology and biochemical examination, we aimed to determine the mechanisms underlying the identified *B. licheniformis*-associated effects. We found that *B. licheniformis* supplementation significantly enhanced the growth performance of Pekin ducks.

Next, we measured common lipid indicators to evaluate the effect of *B. licheniformis* on lipid deposition (Huo et al., 2022; Sah et al., 2022). We found that FAS, LPL, ACC, and ME in the MLB group were higher than that in CON group. The addition of *B. licheniformis* significantly increased lipid absorption, improved lipid transport, and reduced lipolytic activity in adipose tissue. FA are the basic components of all lipids, in mammals, FAS are either provided directly by the diet or synthesized *de novo* from by FAS (Huo et al., 2022; Raab and Lefebvre, 2022). In addition to its key role in energy storage, FAS is actively involved in many other biological processes. These processes include cell division, protein modification, signal transduction, and cell proliferation (May and den, 2021). LPL is an important member of the lipase family, which can promote the hydrolysis of TG, cholesterol, and phospholipids and catalyze the decomposition of TG-rich chylous particles into FFAs and glycerol monoesters to help synthesize fatty acids (Al-Shammari et al., 2022). FFA in serum can reflect the lipolytic activity in adipose tissue (Coras et al., 2022). We also found that the addition of *B. licheniformis* to the diet promotes fatty acid synthesis via enzyme (such as FAS) upregulation, further promoting lipid metabolism in Pekin ducks.

We identified DEGs between the control and experimental groups. KEGG analysis revealed that the identified DEGs were mainly enriched in nutrient metabolism; including fatty acid, glutathione, fatty acid degradation in lipid, and amino acid (glycine, threonine, and serine) metabolism as well as unsaturated FA biosynthesis and FA elongation (Raja et al., 2016; Esteves et al., 2021). During the laying period, *B. licheniformis* increased lipid deposition, greatly enhanced the regulation of fat synthesis-related enzymes, and actively participated in lipid synthesis. There are two main sources of fat in animals, the first is the digestion and absorption of food by the intestine, the other is the body’s own synthesis of fat; liver tissue is an important part of lipid synthesis and metabolism in poultry (Lauby-Secretan et al., 2016; Dwivedi et al., 2020; Pagidipati et al., 2020; Petrelli et al., 2021). In this process, various enzymes involved in liver lipid metabolism play important roles, such as those involved in liver lipogenesis (FAS, ACC, SCD, and ME), liver fat transport (LPL and ACSL5), and liver fat oxidation (ACOX1). The expression levels of FAS, ACC, SCD, ME, LPL, ACSL5, and ACOX1 in the *B. licheniformis* group were significantly higher than those in the CON group, and *HADHB* (gene involved in the degradation of fatty acids) expression was significantly downregulated, indicating that the addition of *B. licheniformis* to diets significantly enhances hepatic fat synthesis, transport, and oxidation in Pekin ducks.

In addition, we found that the DEGs in the *B. licheniformis* group were annotated to multiple signaling pathways, mainly to the PPAR signaling pathway. There are three different PPAR subtypes: PPAR-α, PPAR-β, and PPAR-γ, which co-regulate lipid metabolism, fatty acid degradation, bile acid biosynthesis, ketone body synthesis and degradation, and adipocyte differentiation (Luo et al., 2023; Yang et al., 2024). SCD and ME, upregulated DEGs, were mainly enriched in PPAR-α and PPAR-γ pathways and regulated lipogenesis. LPL and ACSL5, downregulated DEGs, were riched in PPAR-α, PPAR-β, and PPAR-γ pathways. HADHB was downregulated and mainly annotated in the fatty acid degradation pathway. ACOX1 was enriched in PPAR-α, PPAR-β, and PPAR-α pathways and mainly affected the oxidation of fatty acids. This suggests that *B. licheniformis* regulates lipid biosynthesis and fatty acid metabolism via the PPAR pathway (Wang et al., 2024).

Lipidomics is a branch of metabolomics. Lipids are classified into eight groups based on the presence of ketoacyl and isoprenoid groups: fatty acyl groups, glycolipids (GLs), glycerophospholipids (GPLs), sphingolipids (SPs), glycolipids, polyketoacids (derived from condensation of ketoacyl subunits), sterol lipids and isoprenoid lipids. Polyketoacids (derived from the condensation of ketoacyl substituents), sterol lipids, and isoprenoid lipids with fatty acids (FA) included in the fatty acyl group (Yang and Han, 2016). Glycerophospholipids are composed of a group of phosphoric acids and terminal esters X), which, depending on the substituent, such as ethanolamine, choline, serine or inositol, can be subdivided into a number of different subclasses, such as phosphatidic acid, phosphatidylcholine, phosphatidylserine, and phosphatidylinositol, among which phosphatidylcholine (PC), phosphatidylserine (PS), phosphatidylethanolamine (PE), and phosphatidic acid (PA) are known to have proinflammatory properties (Fahy et al., 2011). Glycerophospholipids enable cell membrane recognition of proteins which triggers signaling. Glycerol esters are composed of long-chain acyl groups, alkyl groups, and polar alcohols and can be classified as monoacylglycerol (1 FA), diacylglycerol (2 FA), and triacylglycerol (3 FA) depending on the number of acyl molecules. FAs generally consist of a straight chain of an even number of carbon atoms (also known as an acyl chain) with a hydrogen atom at one end of the chain along the length of the chain and a carboxyl group (-COOH) at the other end (Coras et al., 2022). Serum metabolomic analysis of Pekin ducks fed a *B*. *licheniformis*-supplemented diet revealed a significantly altered lipid metabolism in these ducks, which was mainly manifested in the regulation of glycerophospholipids, fatty acids, and glycerolipid metabolites, suggesting that B. *licheniformis* influences the synthesis and catabolism of glycerophospholipids, fatty acids, and glycerolipids to enhance growth performance in Pekin ducks.

After digestion and absorption of proteins in the intestine, the resulting amino acids are transported via blood to the liver, where protein synthesis and energy metabolism occur (Sikalidis, 2013). Excess amino acids stimulate the release of insulin and inhibit autophagic proteolysis, while simultaneously acting as substrates for other metabolic pathways, such as adipogenesis and gluconeogenesis (Bröer and Bröer, 2017). To maintain metabolic homeostasis, plasma and tissue amino acid levels are efficiently balanced by their uptake, synthesis, and utilization, and can be monitored by several amino acid sensing and signaling pathways (Doi et al., 2017).

One of the best-characterized amino acid-sensing pathways is the conserved amino acid response (AAR) pathway, which is a part of the integrated stress response (ISR) pathway and is activated when amino acids are limited (Harding et al., 2003). In amino acid surplus, amino acid-loaded tRNAs provide amino acids for protein translation in the ribosome, whereas in amino acid deficit, uncharged tRNAs accumulate in the cytoplasm and are recognized by the enzyme GCN2, a serine-threonine kinase (Dong et al., 2000). Binding of GCN2 to these uncharged tRNAs leads to a conformational change, followed by autophosphorylation of GCN2, which triggers phosphorylation of the eukaryotic translation initiation factor eIF2α and inactivation of the eIF2 complex, which in turn inhibits protein synthesis, reducing the need for amino acids. Serine-threonine produces protein kinase, mTORC1, which is a nutrient sensor and a master regulator of metabolism that integrates hormone- and growth factor-induced signals to stimulate anabolism and inhibit catabolism (Liu and Sabatini, 2020).

Glutathione (GSH) is the most abundant intracellular low molecular weight thiol in cells and tissues and plays a crucial role in many cellular processes, including antioxidant defense, regulation of protein function, protein localization and stability, DNA synthesis, gene expression, cell proliferation, and cell signaling. GSH is essential for the maintenance of redox homeostasis in cells and tissues, protects cells and tissues from oxidative and other forms of stress, and is intimately involved in the regulation of redox signaling pathways and detoxification responses (Forman et al., 2009). GSH synthesis requires glutamate, cysteine, and glycine and occurs in two steps: formation of the dipeptide γ-glutamylcysteine from glutamate and cysteine via glutamate-cysteine ligase (GCL, the ATP- and rate-limiting step in glutathione synthesis), followed by the addition of glutathione by the enzyme glutathione synthase glycine (Lu, 2013). Oxidative stress and GSH depletion induces GCL transcription activity (Lu, 2009). Previous studies have shown that the fermentation of white ginseng by *B. licheniformis* increases the total phenolic content where carboxylic acids and their derivatives (glycerophosphorylcholine, amino acids, peptides and their analogs) accounts for a large proportion of the metabolites. The results of the present study confer with these results. DEGs and DEMs were enriched in the GSH, glutamate, cysteine, and glycine metabolic pathways. GSH exhibits strong antioxidant activity, which is also consistent with the findings that *B. licheniformis* contributed to the increase in antioxidant capacity observed in Pekin ducks (Chen et al., 2022).

After combining transcriptomic and metabolomic analyses, we found that *B. licheniformis* affected lipid metabolism in Pekin ducks, which was mainly regulated through lipid and glycolytic metabolism, with lipid metabolism mainly reflecting fatty acid synthesis, catabolism, and elongation, and glycolytic metabolism mainly through glycine, serine, and threonine metabolism. In addition, hydroxytetradecanoyl-CoA and L-palmitoylcarnitine could be potential biomarkers for the *B. licheniformis*-induced regulation of lipid metabolism.

## Conclusion

The addition of *B. licheniformis* to the diet of Pekin ducks significantly altered lipid metabolism via glycerophospholipids, fatty acid, and glycerolipid metabolite regulation. *Bacillus licheniformis* also altered fatty acid synthesis, catabolism and elongation, fat digestion and absorption, as well as glycine, serine, and threonine metabolism. Hydroxytetradecanoyl-CoA and L-palmitoylcarnitine could serve as potential biomarkers for the regulation of lipid metabolism by *B*. *licheniformis*.

## Data Availability

The raw data presented in the study can be found at: https://www.jianguoyun.com/p/DeD6688QruDUDBiKuMYFIAA.

## Abbreviations

B. Licheniformis, *Bacillus* licheniformis; TC, Total cholesterol; TG, Triglyceride; LDL-C, Low density lipoprotein; HDL-C, High density lipoprotein; VLDL, Very low density lipoprotein; FFA, Free fatty acid; FAS, Fatty acid synthetase; ACC, Acetyl CoA carboxylase; ME, Malic enzyme; LPL, Lipoprteinlipase; OPLS-DA, Orthogonal Partial Least Squares Discriminant Analysis; DEMs, Differentially expression metabolisms; DEGs, Differentially expression genes; GO, Gene Ontology; COG, Clusters of Orthologous Groups; KEGG, Kyoto Encyclopedia of Genes and Genomes; WGCNA, Weighted correlation network analysis; MAPK, mitogen activated protein kinase; KGML, KEGG Markup Language; ACSL5, Acyl-CoA Synthetase Long Chain Family Member 5; SCD-1, Stearoyl-CoA desaturase 1; HADHB, Hydroxyacyl-CoA Dehydrogenase Trifunctional Multienzyme Complex Subunit Beta; ACOX1, Acyl-CoA Oxidase 1; ELOVL2, Elongation Of Very Long Chain Fatty Acids-Like 2.
