# Supplementary material for: Multi-omic analysis reveals that Bacillus licheniformis enhances pekin ducks growth performance via lipid metabolism regulation
Source: Front Pharmacol. 2024 Jun 11;15:1412231. doi: 10.3389/fphar.2024.1412231 (PMC11201536; doi:10.3389/fphar.2024.1412231)
Supplement: Supplementary file 1 [file Table1.pdf]

Table S1 Gradient parameters for liquid chromatography

Time（min）	Speed（μL/min）	A%	B%
0.0	400	98	2
0.25	400	98	2
10.0	400	2	98
13.0	400	2	98
13.1	400	98	2
15.0	400	98	2

Table S2 The reaction system of qPCR

Ingredients	用量（μL）
2×mix	5
338F（10uM）	0.4
806R（10uM）	0.4
ROX Dye	0.2
cDNA	1
ddH_2O	Add to 10

Table S3 Sequences for primers of qPCR

Gene	Primers（5'→3'）	bp
β-actin	F: GGTATCGGCAGCAGTCTTA	158
	R: TTCACAGAGGCGAGTAACTT	
ACSL5	F: TGGTATGACTCTGAAGACGGCTGAA	256
	R: TGTTGTCCAGTCTCCAGGCATTGA	
FAS	F: GCTGAGAAACGCCAATACC	181
	R: GAGCAAGACACCGCAAACT	
SCD	F: CACAGCCTTCATCGACTTCA	109
	R: GTGACTCCCATCTCCAGTCC	
HADHB	F: CAGCCGACGAGACCTAAGATTT	125
	R: TAGCTGGGAGGAACTGCTCA	
ACOX1	F: GTGGATATCAACAGCCCCGA	217
	R: GAATCTGGAGGACTTTTTCCG	
ACC	F: AAGGTTATGTGAAGGATGTGGATGA	303
	R: CAATAATCTTCTGATGCCTGCGTT	

ELOVL2	F: GACCTCGAGATGCCAGAGTT	231
	R: AGTTGTAGCCTCCTTCCCAC	
ELOVL3	F: CACTGTCGGTATCCTGGCTT	182
	R: CTCCAACCACTCACTGGCTC	
ELOVL6	F: CCGTGTTGACTTTGCAGGAA	196
	R: CTCAGGGACCAGAGCACTAG	
